# Feasibility and acceptability of involving bilingual community navigators to improve access to health and social care services in general practice setting of Australia

**DOI:** 10.1186/s12913-023-09514-4

**Published:** 2023-05-11

**Authors:** Sabuj Kanti Mistry, Elizabeth Harris, Xue Li, Mark F. Harris

**Affiliations:** Centre for Primary Health Care and Equity, University of New South Wales, Sydney, Australia

**Keywords:** Access to health and social care, Culturally and linguistically diverse, Bilingual community navigator, General practice, Feasibility and acceptability

## Abstract

**Background:**

Patients from culturally and linguistically diverse (CALD) backgrounds often face difficulties in accessing health and social care services. This study explored the feasibility and acceptability of involving community health workers (CHWs) as bilingual community navigators (BCNs) in general practice setting, to help patients from CALD backgrounds access health and social care services in Australia.

**Methods:**

This research was conducted in two general practices in Sydney where most patients are from specific CALD backgrounds (Chinese in one practice and Samoan in other). Three CHWs trained as BCNs were placed in these practices to help patients access health and social care service. A mixed-method design was followed to explore the feasibility and acceptability of this intervention including analysis of a record of services provided by BCNs and post-intervention qualitative interviews with patients, practice staff and BCNs exploring the feasibility and acceptability of the BCNs’ role. The record was analyzed using descriptive statistics and interviews were audio-recorded, transcribed, and thematically analyzed.

**Results:**

BCNs served a total of 95 patients, providing help with referral to other services (52.6%), information about appointments (46.3%), local resources (12.6%) or available social benefits (23.2%). Most patients received one service from BCNs with the average duration of appointments being half an hour. Overall, BCNs fitted in well within the practices and patients as well as staff of participating practices accepted them well. Their role was facilitated by patients’ felt need for and acceptance of BCNs’ services, recruitment of BCNs from the patient community, as well as BCNs’ training and motivation for their role. Major barriers for patients to access BCNs’ services included lack of awareness of the BCNs’ roles among some patients and practice staff, unavailability of information about local culture specific services, and inadequate time and health system knowledge by BCNs. Limited funding support and the short timeframe of the project were major limitations of the project.

**Conclusion:**

BCNs’ placement in general practice was feasible and acceptable to patients and staff in these practices. This first step needs to be followed by accredited training, development of the workforce and establishing systems for supervision in order to sustain the program. Future research is needed on the extension of the intrevention to other practices and culture groups.

**Supplementary Information:**

The online version contains supplementary material available at 10.1186/s12913-023-09514-4.

## Background

The provision of care within the health system in Australia and many other countries is often fragmented [1, 2]. There is a lack of coordination between the different tiers of health services especially between specialist services and general practice [3]. This coordination is particularly important as the population in Australia ages with an increased prevalence of chronic conditions requiring more integrated care to reduce hospitalizations and the concomitant burden to the health system [4–6].

The people from culturally and linguistically diverse (CALD) backgrounds, (defined as being either born overseas, having a parent born overseas, or speaking a variety of languages) comprises 27.6% of the Australian population [7]. This population group faces particular problems accessing and navigating health and social care services in Australia [8]. This is due to a number of factors including their limited health literacy, language and communication problems, lack of information about the local resources contributing to their limited access to health and social care services [9, 10].

Pervious research from overseas has demonstrated the effectiveness of community health workers (CHWs) in helping patients to navigate health services [11–13], preventing hospital admission and readmission [14, 15], reducing burden to health system [16] and promoting self-management [17, 18]. Their roles do not replace those of other providers working in the primary care setting. Rather, they work along with other members of the healthcare team in helping the patient address their barriers to accessing health and social care services. In our recent systematic review [19], we found that CHWs were effective, particularly in increasing screening for chronic disease, reducing hospital admission and readmission, and improving access to primary care services.

Since CHWs are recruited from the same community they serve, they are closely connected with the members of the community and understand the local health system [20, 21]. CHWs are likely to be aware of the available health and social care services and how to navigate those services [22, 23]. These qualities of CHWs enable them to act as a bridge between health and community and social care services [24, 25].

In the previous phase of the research, we interviewed staff from selected general practices in Sydney, New South Wales, Australia, where most of the patients spoke a language other than English at home [26]. Later, we carried out a codesign exercise involving patients, their caregivers, CHWs, and other health providers [27]. The participants of these studies identified barriers faced by the patients from CALD backgrounds in accessing health and social care services and felt that CHWs trained as bilingual community navigators (BCNs) could be a potential way to address these problems and help the patients better access services in general practice setting. This had not been previously tested in Australian general practice except for the role of Aboriginal health workers (AHWs) [28], working in Aboriginal Community Controlled Health Organizations. Therefore, the present study sought to explore the feasibility and acceptability of involving BCNs in the general practice setting in Australia.

## Methods

### Study design and setting

A mixed-method design was employed to explore the feasibility and acceptability of involving BCNs in general practice setting. We approached all five practices that had participated in the previous phase of the research [26]. However, due to demands associated with increased presentations and vaccination in general practices during the COVID-19 pandemic, only two of these practices were able to participate. Both practices had previous long-term collaboration with the research team. In one (practice A) of these practices most patients were of Chinese background, while in other practice (Practice B) major proportion of the patients were of Samoan background. Notably, patients’ backgrounds were determined based on the country from where they migrated.

### The intervention

#### Development of intervention

**Fig. 1:**
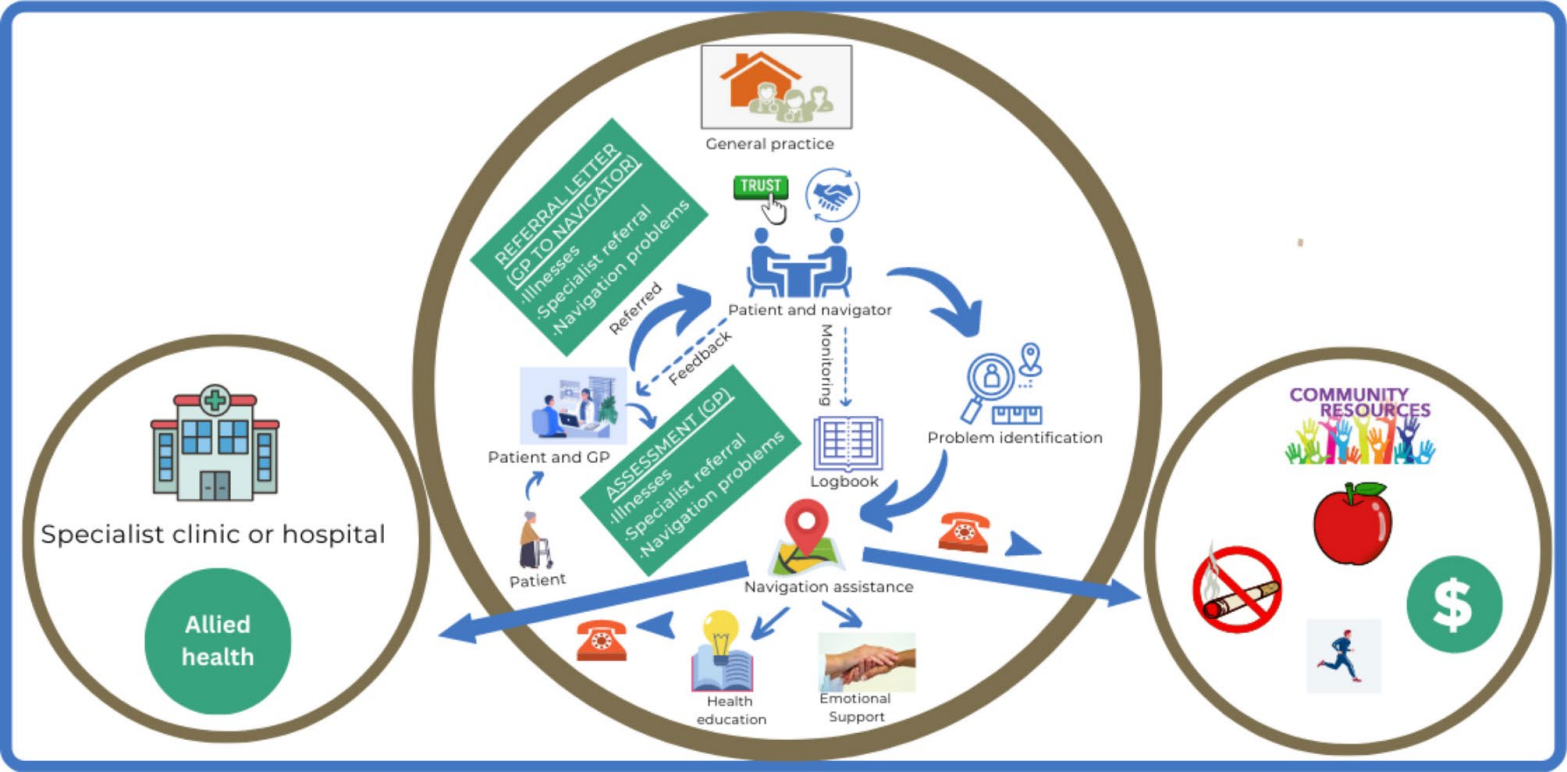
Navigation model of care

Based on the initial scoping exercise [26] and codesign workshops [27] a navigation model of care was developed, in which navigators helped connect patients with health and social care services (Fig. 1). Navigators’ roles would involve addressing patient barriers and helping them better access health and social care services. Development of this model of care was also informed by the teamlet model [29] in which a CHW worked alongside a family practitioner to improve access to health and social care services in the US. In line with the access to health care framework developed by Levesque and colleagues [30], our model of care involved navigators working to both address barriers to access and support patients and their families to acquire the knowledge and skills required for them to navigate the health system.

#### Recruitment and training for BCNs

We developed a job description for BCN and advertised in popular media highlighting our preference for membership of a CALD community and experience of working in the community as well as competencies required to serve the role of a BCN. Short listed candidates were invited for an interview, and finally, we recruited 12 CHWs from five culture groups, namely Bengali (2), Chinese (3), Hindi (2), Arabic (2) and Samoan (3). The CHWs with these culture groups were selected matching the main language groups other than English of patients in the five general practices that participated in the previous phase of the research [26].

The CHWs participated in a blended training for 26 h (4 h face-to-face and 22 h online) during June-September 2021. The training program was developed by three coauthors (SKM, EH, MH) over four months following consultations with other experts from Australia and overseas and was also informed by the structure and main content themes from a review of other patient navigator training programs such ARC Patient Navigator Training [31]. The online training comprised eleven learning modules (Box 1), each with two hours of online learning consisting of notes, a powerpoint presentation, supporting videos, reading materials and a quiz to assess the level of competency gained from the training. The face-to-face training provided a basic orientation on the concepts and roles of healthcare navigation and was facilitated by two coauthors (SKM, EH). The CHWs were paid 50 AUD/hour for undertaking the training, costing a total of 1,300 AUD per participant. All the CHWs actively participated in the training, demonstrating that they had acquired knowledge through their performance in the quiz with an average score of 90%.

##### Box 1: Navigator Training modules

1. Understanding the health system and general practice.
2. Introduction to chronic disease.
3. Preventive health care - risk and protective factors/supporting lifestyle change.
4. Social determinants of health/access to health care.
5. Patient navigators’ roles and responsibilities.
6. Cultural competence.
7. Effective communication and support for self-management.
8. Identifying community resources.
9. Client needs assessment/problem identification.
10. Professional responsibilities and boundaries.
11. Medicine and medication adherence.

#### Procedure

We developed the intervention materials (intervention flyer, patient referral form, BCN working manual, navigation logbook, needs assessment and problem-solving checklist) before three trained navigators were placed in two practices. The navigators were placed in the practice for ten weeks during the period of October to December 2021. They were present in the practice for two sessions a week - each lasting for three hours. They were also allocated an additional two hours in each week if required (e.g., for follow up of patients outside the two sessions). The BCNs were paid on a casual basis for their time in practices.

Patients requiring navigation assistance were identified by the GP who informed them about the BCN and then referred them to the BCN using a referral form (Supplementary file 1). The GP informed the patients that he/she would like to refer them to the BCN. The patient then gave written consent to this and to the evaluation. The BCN met with the referred patients, discussed their needs, priorities, and potential solutions. They completed a needs assessment and problem-solving checklist form and offered navigation assistance to the patients. Besides providing support for navigation, BCNs also provided emotional support to the patients (passionate listening to patient problems and showing empathy towards their problems). However, this did not involve any psychological counselling.

During the intervention, BCNs were asked to complete a logbook which contained detailed information on the tasks undertaken and the time spent with each patient. BCNs also met with the research team and the practice staff fortnightly where they were offered mentoring and emotional support. The research team also maintained regular contact with the practice staff during the intervention to gain insight on the progress of the intervention and resolve any issues that arose.

### Data collection

The logbooks (Supplementary file 2) completed by BCNs were analyzed quantitatively to explore selected demographic and clinical characteristics of the patients and types of services they received.

We carried out post-intervention semi-structured qualitative interviews with 16 participants (patients/caregivers, BCNs and practice staff) (Table 1). We adopted a convenience sampling technique to select patients to interview who were referred by the GPs and subsequently met the BCNs. We developed practice-specific list of patients who participated in the intervention. Patients were then approached over telephone one-by-one from these lists by a research team member (SKM, XL) and were invited to take part in an interview. Consented patients were then interviewed over telephone, and they received a gift voucher for their time. We continued interviewing patients from each practice until saturation of themes was achieved. Similarly, after receiving the informed consent, all three BCNs and three practice staff (two GPs and one practice nurse) who participated in the intervention were interviewed by a research team member over telephone.

**Table 1:** Characteristics of the patients and description of the intervention (N = 95)

Characteristics	n	%
Age (years) Mean: (66.4 ± 15.0)		
< 45	12	12.6
45–69	41	43.2
≥ 70	42	44.2
Sex		
Male	62	65.3
Female	33	34.7
Suffering from non-communicable chronic conditions		
No	43	45.3
Yes	52	54.7
Language spoken at home		
Cantonese	20	21.1
Mandarin	61	64.2
Samoan	14	14.7
Services received		
Help booking appointment with specialist/hospital	50	52.6
Help booking for biochemical test	7	7.4
Help with paperwork	5	5.3
Information about specialist appointment	44	46.3
Information about local resources	12	12.6
Information about social benefit package	22	23.2
Help arranging transport	8	8.4
Fax referral letter to clinic/hospital	33	34.7
Emotional support	15	15.8
Number of services received		
1	47	49.5
2	33	34.7
3 or more	15	15.8
Number of visits		
1	63	66.3
2 or more	32	33.7
Average navigation time (min) Mean: (28.3 ± 15.0)		
< 30	44	46.3
≥ 30	51	53.7

All the participants were provided with the Participant Information Statement and Consent Form (PISCF) and were allowed sufficient time to make their decision to participate in the interviews. Separate semi-structured interview guides were prepared for patients/caregivers, BCNs and practice staff which were piloted before use. SKM conducted interviews in English with all the participants except patients of practice A. Patients from practice A were interviewed in either Mandarin or Cantonese language by XL who is also from Chinese background. Because of the restrictions on face-to-face interactions imposed due to the COVID-19 pandemic (December 2021 to February 2022), all interviews were conducted over telephone. Each interview took between 20 and 60 min. All the interviews were audio-recorded and transcribed. The interviews of the Chinese patients were translated into English language before the transcription.

### Data analysis

All the quantitative information extracted from the navigator logbook were analysed in Stata (Version 14). We used descriptive statistics to compute the frequencies and proportions of the categorical variables. Continuous variables were reported using means and standard deviations.

Qualitative data analysis was carried out using an inductive thematic multistep approach [32]. SKM completed the first three steps: (1) an initial read of the transcripts to understand the data; (2) deeper reading of the transcripts and identifying initial codes; (3) identified codes were then grouped into key themes. In the fourth step, initial themes were refined by SKM to provide better representation through discussion among the research team members (MFH, EH, XL). Next, the themes were named, and categorized into subthemes where necessary. Finally, the data were thematically analysed and a narrative was written. Data analysis was undertaken using an inductive approach based on themes identified in the previous phases of this research [26, 27] which were not consistent with established frameworks such as the theoretical domains framework for behaviour change [34]. Data were managed in the NVivo (Version12.0). Qualitative methods are reported (Supplementary file 3) according to COnsolidated criteria for REporting Qualitative studies (COREQ) guidelines [35].

### Ethics statement

Ethical approval was obtained from the UNSW Human Research Ethics Committee (HC210529) before conducting the study. All the participants including patients/caregivers, BCN and practice staff provided their full informed consent to participate in the research.

## Results

The characteristics of the patients and services they received was extracted from the navigation logbook (Table 1). A total of 95 patients (80 from the practice A and 15 from practice B) participated in the intervention, most of whose were aged 65 years and above (66.3%), female (65.3%), speaking Mandarin at home (64.2%) and was suffering from non-communicable chronic conditions (54.7%). BCNs helped more than half of the patients (52.6%) in booking their appointments with specialists or hospital. Similarly, around half of the patients (46.3%) received information about specialist appointments such as what to bring with them to their appointment and any biochemical tests to be completed before the appointment. On some occasions, while booking appointments for the patients, BCNs were informed by the receptionists of clinics/hospital that they had not received the referral form sent by the GP. BCNs, therefore, re-sent patient referral forms via fax to the designated clinic/hospital for around one third (34.7%) of the patients. The BCNs also informed some patients about the local resources, i.e., local preventive services such as physical activity or diabetes education groups (12.6%) or available social benefits (23.2%). Nearly one-quarter of the patients received emotional support from the BCNs, and a few patients also received help with paperwork i.e., filling forms such as specialist appointment form, transportation, and accessing pathology collection centres (if referred by GP). Half of the patients received one service, while 34.7% received two services and 15.8% received three or more. Most of the patients had one encounter with BCNs (66.3%) with an average duration of appointment with BCNs being an average of half an hour.

### Feasibility and acceptability for BCNs’ involvement

The feasibility and acceptability of the intervention was determined through in-depth interviews undertaken post-intervention with selected patients, practice staff and BCNs. The summary of the participants are presented in Table 2.

**Table 2:** Summary characteristics of the participants

ID	Code	Gender	
1	GP	M	
2	GP	F	
3	PN	F	
4	BCN	F	
5	BCN	F	
6	BCN	M	
7	Patient	F	
8	Patient	M	
9	Patient	F	
10	Patient	F	
11	Patient	M	
12	Patient	F	
13	Patient	M	
14	Caregiver	F	
15	Patient	F	
16	Patient	F	

GP: General Practitioner; BCN: Bilingual Community Navigator; PN: Practice Nurse

Overall, it was revealed that the involvement for BCNs was feasible in the general practice setting and that they were well accepted by the patients and practice staff. We have grouped the issues identified by the participants under four topic headings: (1) Contribution of BCN roles to addressing patient care problems; (2) Facilitator for BCNs’ roles in helping patients navigate services; (3) Barriers for BCNs’ roles in helping patients navigate services; (4) limitations of the intervention.

#### Contribution of BCN roles to addressing patient care problems

##### **Overcoming communication problems to addressing patient care problems**

Many patients described their difficulties in booking an appointment with the hospital or specialist services and that practice staff had been too busy to help them. Patients especially mentioned their inability to communicate when they approached the health services over phone for an appointment because of their poor English language literacy. They also found it difficult to communicate on the phone to make an appointment with a medical specialist or hospital as this often required several steps before the call reached the right person. Often referral letters sent by the GP did not reach the services, referrals remained unattended, patients remained unaware of the booking (forget or do not open their letter box to get the referral letter sent to them) or there was no follow up after the referral appointment. BCNs helped the patients booking the appointment through calling the appropriate services, resending the referral letter, or following up after the booking. They also helped arrange interpreters for some patients to overcome their communication barriers faced during their referral consultations.

“*Oh, you know, my English is poor, so no English, it is very difficult to call the public hospitals or clinics, sometimes they don’t even answer*” (Patient, participant 9).

##### **Improving health system literacy**

Participant reported that patients from the CALD backgrounds had limited understanding of the health system in Australia. In response, BCNs explained to patients how the Australian health system worked, how to book appointments, what preparations were required to attend an appointment and the tasks they needed to complete after the appointment as instructed by the health providers.

“*The doctor, after the check, you know, and they have such a long queue, and email all that. We don’t understand, so…there is a person (BCN) to do these for us*” (Patient, participant 12).

##### **Building a trusted relationship between patients and BCNs**

Language and cultural similarities with that of the patients, as well as their interpersonal skills, helped BCNs to build a trusted relationship with the patients. Many patients also appreciated the patience shown by BCNs in listening to their problems. Practice staff also mentioned the ability of BCNs to develop a trusted relationship with patients. They praised the good personal qualities of BCNs in this regard.

“*We were very lucky to have this BCN. He’s got excellent rapport. So, I think that’s really important to have a navigator that has a sense of presence and has a sense of leadership and has a sense of just an ability to communicate. So that was really, really good to get from him.*” (GP, participant 2).

Yet, a few patients did not accept BCNs’ services and this being a barrier to their effectiveness.

“*He (patient) thinks, oh I’m Australian-born Samoan. He (BCN) doesn’t know how to speak the language. I’m not going to talk to him (BCN) because he’s below me.*” (BCN, participant 6).

##### **Helping to identify and access local resources**

Many participants noted that patients lacked information about the local preventive health services (i.e., local physical activity groups, language specific dieticians etc.) and practice staff lacked the time to identify those services and help patients navigate to other services. As part of their role, BCNs identified available local resources and helped patients access those services. BCNs were especially helpful as they provided this information to the patients in their own language.

“*I think that navigator can provide them more information about community resources in their languages, so they will be able to access the community resources, for example, like community transport.*” (BCN, participant 5).

#### Facilitators for BCNs’ roles in helping patients navigate services

##### **Perceived need for the services**

The major facilitator of the BCN roles was the strong felt need for the service. Most of the participants acknowledged that patients needed the service and thus accepted BCNs roles. They found patients were relaxed discussing their problems with BCNs and asking for help in addressing those problems.

“*This service (BCN) is very important for people like us who don’t speak English well, sometimes all the specialists only speak English. Sometimes the doctors don’t have translation, then you waste time on finding translators. Sometimes there is telephone translation, but it’s difficult still.*” (Patient, participant 9).

##### **Acceptability of BCN roles to patients**

Many patients talked about their language barriers in making the appointment or accessing other service and acknowledged BCNs’ role in addressing their language barriers and helping them better access health and social care services.

“*I don’t know English. I thought there must be a way out if I just got there. Really there was. Somebody did it for me. In the last I had the check. I would just wait in home if it wasn’t for the call, she made for me.*” (Patient, participant 15).

“*It is very helpful for the elders like us. It’s worse on bad Chinese. Some elders can’t even speak Chinese well. You know, there are some elders who don’t have much knowledge and education. They must make appointment on their own and deal with the hospital. It would be more difficult, right? So, I think this is good.*” (Patient, participant 9).

##### **Qualities of BCNs**

Several participants mentioned the importance of BCNs being selected from the same cultural and language background of the patients. Participants mentioned that the patients felt very comfortable talking to BCNs as they were also from their cultural and language background.

“*Because he sometime, he talks a lot about in the home language, so I understand what he means.*” (Patient, participant 8).

BCNs took time listening to patient problems, exhibited communication skills, leadership qualities and link to the community. These qualities helped them to develop rapport with patients and to provide their services more effectively.

“*Yeah, I was really calm and listened to when she says it. But the same time I, yeah, I can put myself into her shoes as well. That’s probably why she started to talk to me.*” (BCN, participant 4).

BCNs also mentioned that the training they received before the intervention was helpful for them to effectively serve their roles. They mentioned that several components of the training program such as information on health system, medical knowledge, language and cultural competence, and communication skills helped them to build the competencies required to help address the problems faced by the patients. Practice staff also felt that BCNs received sufficient training to work independently with minimum supervision.

“*Actually yes…. (training)…. was very useful, like the cultural and languages and the communication technique. Also, some of the medical terms - medical knowledge training that is really helpful…….*” (BCN, participant 5).

All these qualities by BCNs helped them develop rapport with patients and this was established in brief conversations with patients about their everyday life, their previous health service experience, and the problems they faced in accessing care. BCNs offered a non-judgmental ear for the patients to speak about their problems.

##### **BCNs benefitted from their work**

BCNs were enthusiastic and motivated about their roles. They found the role rewarding as they were able to listen to the problems of many patients from their own community and was able to help them. They felt happy and fulfilled being able to serve their community.

“*So, I’ve been grateful, like, meeting people and listen to their life stores. That will be like, quite happy and brand-new experience to me. I’m very grateful of this opportunity, so thank you.*” (BCN, participant 5).

##### **Organizational fit and contribution by BCNs**

Both practices were able to offer a separate place for the BCNs within the practice where they could consult with patients helping them access their desired services. The work environment was also comfortable for the BCNs, and they felt that they received adequate support from the practice staff such as doctor, practice nurse or receptionist when it was required.

“*I don’t think there will be a problem with us in the practice having problems getting them to fit in. So, if there was, they could always talk to either myself or a nurse, someone else in the team.*” (GP, participant 1).

Most participants believed that BCNs contributed to improving the efficiency of care at the practices. They mentioned that BCNs’ role facilitated patient appointments with the specialist care or hospital, helped make the patient more aware of how health system works, helped the patient effectively locate and navigate the local services, and prepared patients to self-manage their problems.

“*I think that they (BCNs) will see they (patients) get the appointment time quicker and easier.*” (GP, participant 1).

Practice staff also mentioned that the roles played by BCNs, which otherwise would have been played by other practice staff, helped to reduce their stress and workload.

#### Barriers for BCNs’ roles in helping patients navigate services

##### **Inadequate communication between patients, BCNs and practice staff**

Some patients mentioned that they were quite unaware that BCNs worked in the practice. BCNs also mentioned that they received some patients who didn’t know why they were being asked to see the BCN. While practice staff mentioned that they had been oriented to the new role by BCNs, they also acknowledged that not all practice staff participated in the intervention, and many were still unaware of BCNs role in the practice.

“*The GP said wait outside, there would be a woman (BCN) to call you in. We don’t know what it meant. How to help, what’s happening. Then we went in and talked to her a little while. Then we learned that it was an organization of something.*” (Patient, participant 13).

While most participants felt it was important for patients to be referred to BCNs by GPs, they also reported that patients needed to be better screened by GPs before they are referred to BCNs. This is particularly important in busy practices as otherwise the workload might overwhelm BCNs and mean that they would have less time with each patient and increase the waiting time to be seen by the BCNs.

“*I feel that the GP referred everyone to her (the BCN), you know, made her do everything. We waited for a long time before she came…….I have to wait for two elders (in the queue) before it was our turn. It was a really long waiting*.” (Caregiver, participant 14).

One BCN mentioned that it would have been better if the GP provided some more information about the patients in the referral letter and clearly identified the services they required.

##### **Inadequate health and system level literacy by BCNs**

Another barrier identified by many participants was the BCNs’ lack of sufficient prior health and system level literacy by BCNs. Most BCNs had little or no health background and therefore were not aware of some medical terms which made it difficult for them to clearly interpret patients’ problems at times or meet the expectations of other staff. While navigator training program helped BCNs improve their health and health system literacy, it was perceived that still they needed more training especially regarding medications.

“*Maybe they (BCNs) don’t understand very well about some – the medical things. So, they cannot explain clearly to the patients. Maybe that’s one of the barriers.*” (Practice Nurse, participant 3).

#### Limitations of the intervention

##### **Difficulty identifying culture specific referral options**

The GP and BCN working in the Samoan practice mentioned that there was a lack of information about the specialists or other social care service providers who were from Samoan background. They found it very difficult to help patients to find their way to culture specific services as there was no database of service providers from Samoan background.

“*So, there was very little services in our own language (Samoan). That was the first thing that we noted, or we didn’t know where to look. So, a bit of thought to put out there to our researchers is we probably need to do a bit more groundwork to find out.*” (GP, participant 2).

##### **BCN short-term availability**

A major limitation of the intervention was that BCNs were able to be present in the practice for only a limited time on a casual basis because of the limited funding. This made it difficult for GPs to refer the patients to BCNs when they were not present in the practice. It also made it difficult for the BCNs to follow up the patients when they were not present in the practice, but the patients required the help. Moreover, because of limited time in the practice, the BCNs were very busy.

“*So many patients, they don’t know, or they come here and the navigator already gone*.” (Practice Nurse, participant 3).

##### **Discontinuity of the service**

Most of the participants mentioned about the importance of having the BCNs longer-term. They felt the project too short and urged for more funding to continue the project.

“*I think it’s a bit early. It was a pilot study, and it was really just introductions. So had we been able to keep him longer…….to make a proper assessment, but I think the groundwork was started*.” (GP, participant 2).

Because of the short timeframe of the project, BCNs were not able to follow-up patients.

## Discussion

This is the first research exploring the feasibility and acceptability of involving CHWs as BCNs in general practice settings in Australia. Three BCNs were placed in two general practices in Sydney to help patients from CALD backgrounds to better access health and social care services. We found the role was feasible and well accepted by patients and staff of the participating practices.

In line with findings of other studies, BCNs were able to help patients address their communication barriers through helping them booking appointments, improving access to care, and providing education to improve health and system level literacy [36–38]. Notably, most of the patients who received services from the BCNs were aged over 65 years and/or were suffering from chronic conditions. Previous research conducted overseas also documented the importance of navigation support for patients suffering from chronic diseases such as diabetes, cardiovascular diseases, chronic obstructive pulmonary disease (COPD) and cancer [36, 39].

BCNs’ roles also helped address some inadequacies of health system which contribute to inadequate healthcare access by patients from CALD backgrounds. These include culturally incompetent health service delivery and under representation of people from CALD communities within the health workforce [40, 41]. BCNs contributed to addressing these problems and helped patients better access health and social care services [26]. Moreover, BCNs helped reduce the workload of practice staff and patients’ family members. Previous research has also documented the positive impact of patient navigators on the workloads of other health providers such as GPs and practice nurse [37, 42] and the stress on patients’ family members through helping the patients better access services which otherwise would have to be solely done by patients’ family members [43].

As reported in other international research and previous phase of our research [19, 26, 27], recruitment of BCNs from the same culture and language group as patients played a role in developing rapport with patients [44, 45]. CHWs’ good interpersonal skills, patience and identification with their own community has been reported in other studies [44, 46] and was key factors for them to better serve in their roles.

**Fig. 2:**
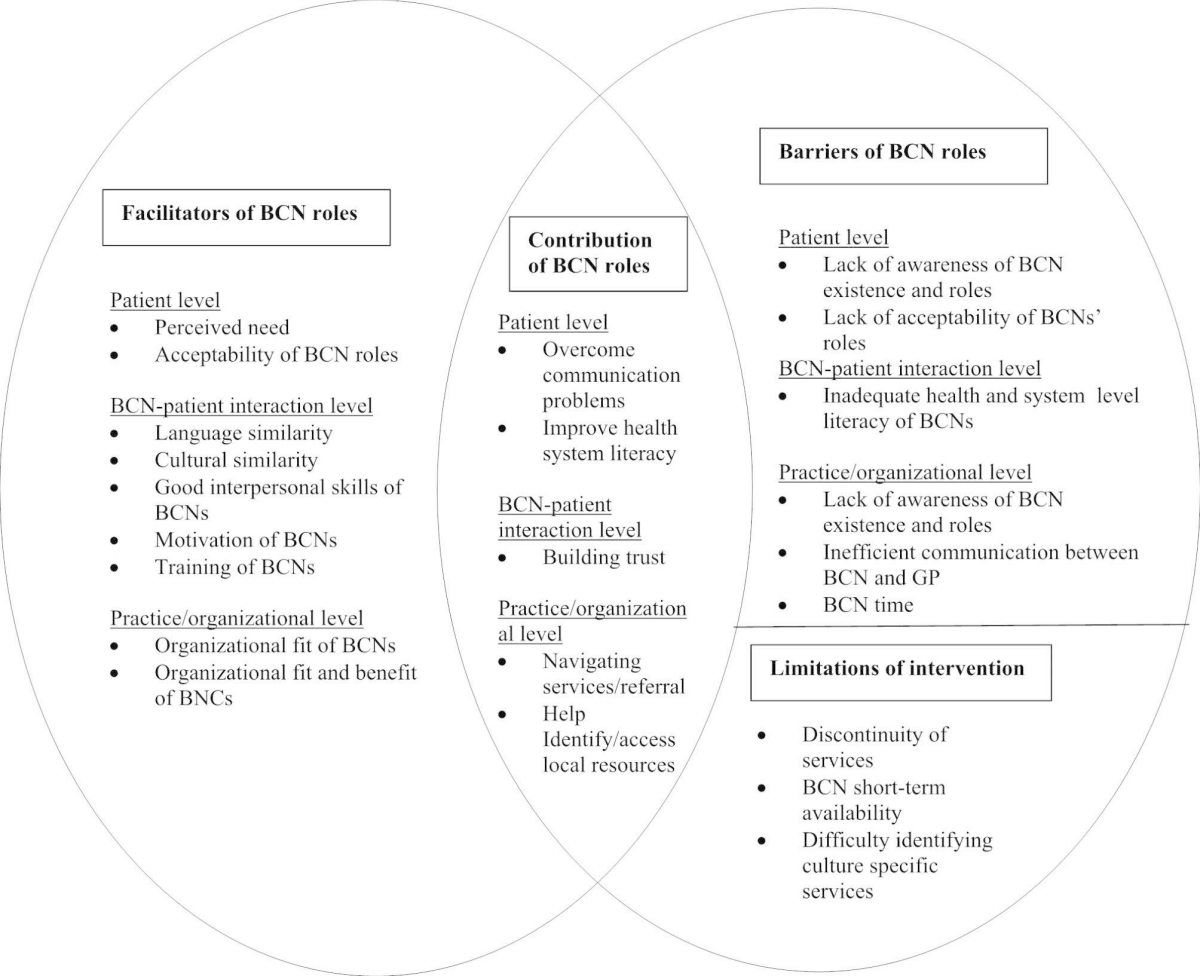
Feasibility and acceptability of the intervention

The contribution by BCNs’ roles in overcoming the patient problems in accessing health and social care services and the concomitant facilitators and barriers of their roles are summarized in Fig. 2. This diagram is underpinned by a number of theoretical frameworks. First, as noted in the methods, the role of the BCNs was informed by the Levesque et al. access to health care model [30]. This helps in our understanding of the importance of CHWs both addressing access barriers within the services and helping to build consumer abilities. Secondly, Fig. 1 emphasises the importance of BCNs intervening at multiple levels of the socio-ecological model [33] - the patient, service delivery and organisational or system levels.

We found that BCNs’ roles were accepted by the patients which has also been supported by the findings of previous research [47, 48]. BCNs’ roles were widely accepted among the patients as there was a perceived need for these services among them[8–10]. Cultural and language similarities with patients and interpersonal skills by BCNs played a crucial role in helping patients to accept them as a trusted service provider [44–46]. The motivation by BCNs towards their role has been recognized as an important facilitator [46]. Moreover, in line with previous studies [49–51], we found that comprehensive training helped them to work effectively with practice staff.

BCNs were also well accepted within the practice as a care team member. Previous systematic reviews exploring the involvement of patient navigators in the primary care setting have confirmed that they were well integrated with primary care teams [37, 52]. The placement for BCNs within the practice also helped to improve patient-provider interactions within the practice [53, 54]. Practice staff also accepted their roles as they were able to independently work with minimum supervision while maintaining the highest level of confidentiality and privacy required in a clinical setting [55, 56].

However, some barriers were noted by the participants for the effective implementation of BCN’s roles. One of the major barriers reported by some participants was the lack of awareness for BCNs’ existence and roles among the patients and practice staff. Previous research also documented the importance of making the patients and practice staff aware of CHWs and their roles as navigators [57, 58]. Moreover, BCNs’ inadequate medical and health system knowledge sometimes acted as a barrier, emphasizing the importance of training [36, 37]. Nevertheless, these barriers were only pointed by a few participants.

BCNs experienced difficulty in finding information about culture specific health and social care services in the local area. While social prescribing has received more attention recently in Australia [59, 60], one of the major limitations to its effective implementation is the lack of information about the culture specific health care services and providers, particularly for Pacific population in Australia [61]. Development of an online database of culture specific providers and local resources similar to that developed in Ontario, Canada may be useful [62].

BCNs were only placed in practices for a limited number of hours per week and this increased the waiting time for the patients. This may be improved by streamlining the referral by GPs and clarification of which patients benefit most from seeing the BCN (i.e., suffering from multiple conditions, requiring frequent visits to health services or having no one in the family to support). This might be flagged by audit or record review. Previous research has also found that where navigators have high workloads, this results in frustration and burnout among the navigators and dissatisfied patients [63, 64]. Likewise, the shorter project duration was a major limitation. Previous research exploring the implementation of navigators has also identified the importance of sustainable funding [46, 65].

### Implications for policy and practice

BCNs were perceived to be useful in addressing some of the problems faced by patients in accessing care. However, their role needs to be supported by adequate training, briefing of all practice staff and funding to employ them over the longer term. Potentially, Medicare could support BCNs as an established workforce and allocate sustainable funding through Team Care Arrangement, as is currently the case for Aboriginal Health Workers [66]. Aboriginal Health Workers play an important role in addressing the needs of the Aboriginal population in Australia through health education, clinical support and brokering culturally appropriate care [28]. As we found in our systematic review [19], other recent studies conducted overseas have documented the effectiveness and cost-effectiveness of CHW-led interventions [67–69] in improving access to services. This suggests the need to scale-up the BCN intervention. Moreover, with current shortages in the clinical workforce in primary care settings in Australia [70, 71], BCNs could provide culturally sensitive care and complement the roles of other practice staff.

Developing and maintaining a trained workforce for this role and providing professional supervision are other important next steps. Health services or non-government organizations could play a role in delivering this as they have done with peer support workers. In the US, support by public health organizations and a consensus on core competencies has supported arrangements for training and supervision [72].

### Limitations of the study

The major limitation of the research was its restrictions to two practices. We explored the feasibility and acceptability of the intervention using qualitative interviews with only 3 BCNs, 3 practice staff and 10 patients. While thematic saturation was reached, this represents a small proportion of the 95 patients who received the intervention. Further evaluation research is needed based on frameworks such as RE-AIM (Reach, Adoption, Implementation and Maintenance) [73]. The intervention was carried out with two cultural groups. Therefore, the findings cannot be generalized for other culture groups. Moreover, our intervention was only three months duration, and thus we were unable to assess long term impacts and sustainability.

## Conclusion

Overall, we found that the intervention was feasible in two selected Australian general practices and was acceptable to patients and staff of these practices. However, not all patients and practice staff were aware of the roles of BCNs and there were significant barriers yet to be overcome. Future large-scale research is needed to explore the impact of engaging BCNs in general practices with other cultural background and systems for training and supervision at scale need to be developed. Moreover, future research is needed to further evaluate the balance of cost and benefits of the BCN model of care and unpack the impact of cultural factors on the extent to which patients and their families are able to access and benefit from health and social care services.

## Electronic supplementary material

Below is the link to the electronic supplementary material.

Supplementary Material 1

Supplementary Material 2

Supplementary Material 3

## Data Availability

The datasets generated and/or analyzed during the current study are not publicly available due the organizational policy of the institution undertaking the research but are available from the corresponding author on reasonable request.
