# Correction to: Feasibility and acceptability of involving bilingual community navigators to improve access to health and social care services in general practice setting of Australia

**DOI:** 10.1186/s12913-023-09903-9

**Published:** 2023-08-29

**Authors:** Sabuj Kanti Mistry, Elizabeth Harris, Xue Li, Mark F. Harris

**Affiliations:** Centre for Primary Health Care and Equity, University of New South Wales, Sydney, Australia

**Correction to: BMC Health Services Research (2023) 23:476.**

10.1186/s12913-023-09514-4.

Following publication of the original article [1], the authors identified errors in some of the references. The corrected references are listed below and the original article [1] has been corrected.

- The correct citation of reference #3 should be:

Hall J. Australian Health Care-The Challenge of Reform in a Fragmented System. N Engl J Med. 2015;373(6):493 − 97.

- The correct citation of reference #4 should be:

McPake B, Mahal A. Addressing the Needs of an Aging population in the Health System: The Australian Case. Health Syst Reform. 2017;3(3):236–247.

- The correct citation of reference #5 should be:

Van Gaans D, Dent E. Issues of accessibility to health services by older Australians: a review. Public Health Rev. 2018;39(1):1–16.

- The correct citation of reference #6 should be:

Calder R, Dunkin R, Rochford C, Nichols T. Australian health services: too complex to navigate. A review of the national reviews of Australia’s health service arrangements. Australian Health Policy Collaboration, Policy Issues Paper No. 1 2019. Melbourne: Australian Health Policy Collaboration; 2019.

- The correct citation of reference #11 should be:

Hunter JB, de Zapien JG, Papenfuss M, Fernandez ML, Meister J, Giuliano AR. The impact of a promotora on increasing routine chronic disease prevention among women aged 40 and older at the US-Mexico border. Health Educ Behav. 2004;31(4_suppl):18S–28S.

- The correct citation of reference #12 should be:

Jandorf L, Cooperman JL, Stossel LM, et al. Implementation of culturally targeted patient navigation system for screening colonoscopy in a direct referral system. Health Educ Res. 2013;28(5):803–15.

- The correct citation of reference #13 should be:

Dudley DJ, Drake J, Quinlan J, Holden A, Saegert P, Karnad A, Ramirez A. Beneficial effects of a combined navigator/promotora approach for Hispanic women diagnosed with breast abnormalities. Cancer Epidemiol Biomarkers Prev. 2012;21(10):1639–44.

- The correct citation of reference #15 should be:

Burns ME, Galbraith AA, Ross-Degnan D, Balaban RB. Feasibility and evaluation of a pilot community health worker intervention to reduce hospital readmissions. Int J Qual Health Care. 2014;26(4):358–65.

- The correct citation of reference #17 should be:

Heisler M, Spencer M, Forman J, et al. Participants’ assessments of the effects of a community health worker intervention on their diabetes self-management and interactions with healthcare providers. Am J Prev Med. 2009;37(6):S270–S279.

- The correct citation of reference #18 should be:

Egbujie BA, Delobelle PA, Levitt N, Puoane T, Sanders D, van Wyk B. Role of community health workers in type 2 diabetes mellitus self-management: A scoping review. PloS One. 2018;13(6):1–18.

- The correct citation of reference #26 should be:

Mistry SK, Harris E, Harris MF. Scoping the needs, roles and implementation of bilingual community navigators in general practice settings. Health Soc Care Community. 2022;30(6):e5495–e5505.

- The correct citation of reference #27 should be:

Mistry SK, Harris E, Harris MF. Learning from a codesign exercise aimed at developing a navigation intervention in the general practice setting. Fam Pract. 2022;39(6):1070–1079.

- The correct citation of reference #42 should be:

Health Education England. Care navigation: A competency framework. England: NHS; 2016.

- The correct citation of reference #61 should be:

South Western Sydney Local Health District. Pacific Communities Health Needs Assessment. Sydney: SWSLHD; 2019.

- The correct citation of reference #62 should be:

InformOntario. Ontario 211 Services. 2023. https://informontario.on.ca/our-members/ontario-211-services. 23 Jun 2023.

- The reference #63 in the published paper was in error. It should be removed and replaced with the following citation:

Henderson S, Kendall E. ‘Community navigators’: making a difference by promoting health in culturally and linguistically diverse (CALD) communities in Logan, Queensland. Aust J Prim Health. 2011;17(4):347–54.

## References

[1] Mistry SK, Harris E, Li X, Harris MF (2023). Feasibility and acceptability of involving bilingual community navigators to improve access to health and social care services in general practice setting of Australia. BMC Health Serv Res.

