# Supplementary figures and images for: Feasibility and acceptability of involving bilingual community navigators to improve access to health and social care services in general practice setting of Australia
Source: BMC Health Serv Res. 2023 May 11;23:476. doi: 10.1186/s12913-023-09514-4 (PMC10174608; doi:10.1186/s12913-023-09514-4)

Supplementary file 1: Referral form template


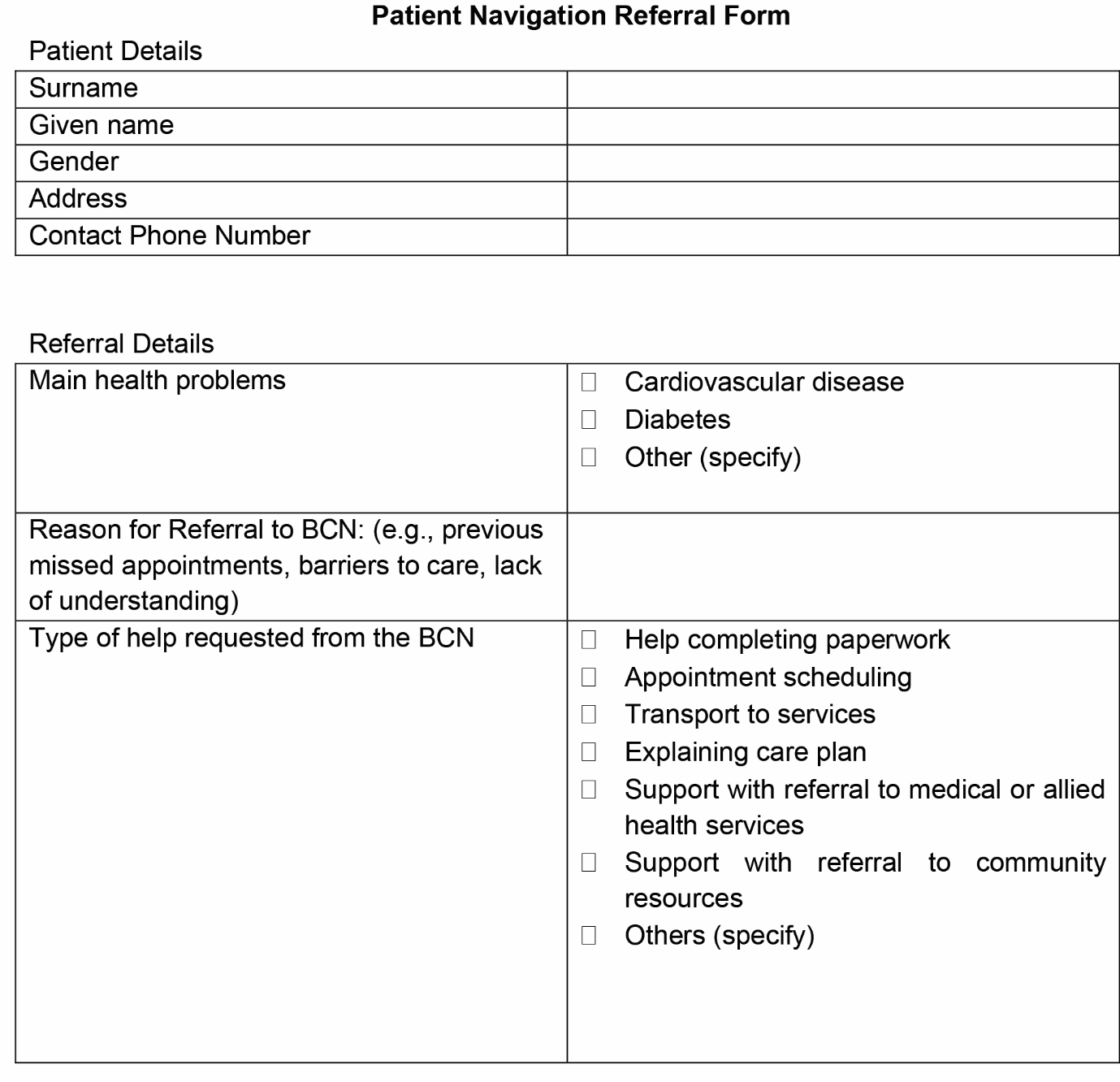

Supplement: Supplementary file 1 — Supplementary Material 1 [file 12913_2023_9514_MOESM1_ESM.docx]
