# Supplementary material for: Feasibility and acceptability of involving bilingual community navigators to improve access to health and social care services in general practice setting of Australia
Source: BMC Health Serv Res. 2023 May 11;23:476. doi: 10.1186/s12913-023-09514-4 (PMC10174608; doi:10.1186/s12913-023-09514-4)
Supplement: Supplementary file 2 — Supplementary Material 2 [file 12913_2023_9514_MOESM2_ESM.docx]

Supplementary file 2: Navigator logbook template

| Patient no. | Patient name | Patient  gender | Patient age | Visit  no | Date | Patient name | Age of the patient | Language/cultural background of the patient and if interpreter needed | Disease suffering  from | Issues identified by GP | Services offered | Key Issues identified by navigator | Services offered | How long did navigation last with patient | | Does the patient have a carer | What type of carer (relative, other) | Patient contact |
| --- | --- | --- | --- | --- | --- | --- | --- | --- | --- | --- | --- | --- | --- | --- | --- | --- | --- | --- |
|  |  |  |  |  |  |  |  |  |  |  |  |  |  | Hour | Min |  |  |  |
| 1 |  |  |  |  |  |  |  |  |  |  |  |  |  |  |  |  |  |  |
| 2 |  |  |  |  |  |  |  |  |  |  |  |  |  |  |  |  |  |  |
| 3 |  |  |  |  |  |  |  |  |  |  |  |  |  |  |  |  |  |  |
| 4 |  |  |  |  |  |  |  |  |  |  |  |  |  |  |  |  |  |  |
