# Supplementary material for: Feasibility and acceptability of involving bilingual community navigators to improve access to health and social care services in general practice setting of Australia
Source: BMC Health Serv Res. 2023 May 11;23:476. doi: 10.1186/s12913-023-09514-4 (PMC10174608; doi:10.1186/s12913-023-09514-4)
Supplement: Supplementary file 3 — Supplementary Material 3 [file 12913_2023_9514_MOESM3_ESM.docx]

Supplementary file 3: COnsolidated criteria for REporting Qualitative studies (COREQ)

| **Item number** | **Guide questions/description** | **Comment** | **Reported on page number** |
| --- | --- | --- | --- |
|  | **Domain 1: research team and reflexivity** | | |
|  | ***Personal characteristics*** | | |
| 1. Interviewer/facilitator | Which author/s conducted the interview or focus group? Interviewer/facilitator | SKM and XL conducted the interview. | 6 |
| 1. Credentials | What were the researcher’s credentials? E.g. PhD, MD | MFH is MD, EH and XL are PhD and SKM is a PhD candidate. | N/A |
| 1. Occupation | What was their occupation at the time of the study? | All authors except SKM are researchers working at the Centre for Primary Health Care and Equity (CPHCE), University of New South Wales (UNSW), Sydney, while SKM is a PhD candidate at the CPHCE, UNSW Sydney at the time of the Study. | N/A |
| 1. Gender | Was the researcher male or female? | Two male identifying and two female identifying. | N/A |
| 1. Experience and   training | What experience or training did the researcher have? | All research team  members have extensive  experience of conducting  qualitative health research | N/A |
|  | ***Relationship with participants*** | | |
| 1. Relationship   established | Was a relationship established prior to study commencement? | Authors had an ongoing collaborations with the general practices participated in the intervention. | 4 |
| 1. Participant knowledge of the interviewer | What did the participants know about the researcher? e.g. personal goals, reasons for doing the research | Participants were provided with the Participant Information Statement and Consent Form (PISCF) and allowed sufficient time to make their decision to participate in the interviews. | 6 |
| 1. Interviewer characteristics | What characteristics were reported about the interviewer/facilitator? e.g. Bias, assumptions, reasons and interests in the research topic | The interviewers (SKM and XL) had an extensive experience conducting qualitative interviews and were significantly oriented about the research before administering the interviews. | N/A |
|  | **Domain 2: study design** | | |
|  | ***Theoretical framework*** | | |
| 1. Methodological orientation and Theory | What methodological orientation was stated to underpin the study? e.g. grounded theory, discourse analysis, ethnography, phenomenology, content analysis | Thematic analysis was undertaken in the research. | 6 |
|  | ***Participant selection*** | | |
| 1. Sampling | How were participants selected? e.g. purposive, convenience, consecutive, snowball | Convenience sampling was adopted to recruit the participants. | 5 |
| 1. Method of approach | How were participants approached? e.g. face-toface, telephone, mail, email | Patients were approached over telephone. | 5 |
| 1. Sample size | How many participants were in the study? | A total of 16 participants participated in the study. | 5 |
| 1. Non-participation | How many people refused to participate or dropped out? Reasons? | A number of participants  did not respond to the  invitation to participate,  however, any participant  did not drop out once agreed to take part. | N/A |
|  | ***Setting*** | | |
| 1. Setting of data collection | Where was the data collected? e.g. home, clinic, workplace | Data collection was conducted over telephone | 5-6 |
| 1. Presence of nonparticipants | Was anyone else present besides the participants and researchers? | None | N/A |
| 1. Description of sample | What are the important characteristics of the sample? e.g. demographic data, date | Presented in result section | 6-7 |
|  | ***Data collection*** | | |
| 1. Interview guide | Were questions, prompts, guides provided by the authors? Was it pilot tested? | Semi-structured piloted interview guides were used. | 6 |
| 1. Repeat interviews | Were repeat interviews carried out? If yes, how many? | No | N/A |
| 1. Audio/visual recording | Did the research use audio or visual recording to collect the data? | All the interviews were audio-recorded and transcribed. | 6 |
| 1. Field notes | Were field notes made during and/or after the interview or focus group? | Interviewers written brief notes after each interview about the interview | N/A |
| 1. Duration | What was the duration of the interviews or focus group? | Each interview took around 20-60 min. | 6 |
| 1. Data saturation | Was data saturation discussed? | Yes | 5 |
| 1. Transcripts returned | Were transcripts returned to participants for comment and/or correction? | No | N/A |
|  | **Domain 3: analysis and findings** | | |
|  | ***Data analysis*** | | |
| 1. Number of data coders | How many data coders coded the data? | Four coders coded the data. SKM initially coded the data and these were discussed with all team members. | 6 |
| 1. Description of the coding tree | Did authors provide a description of the coding tree? | Yes | 6 |
| 1. Derivation of themes | Were themes identified in advance or derived from the data? | Yes, four themes derived from the data. | 7 |
| 1. Software | What software, if applicable, was used to manage the data? | NVivo (Version12.0) was used to manage the data. | 6 |
| 1. Participant checking | Did participants provide feedback on the findings? | No | N/A |
|  | ***Reporting*** | | |
| 1. Quotations presented | Were participant quotations presented to illustrate the themes / findings? Was each quotation identified? e.g. participant number | Yes, presented in Results section | 7-18 |
| 1. Data and findings consistent | Was there consistency between the data presented and the findings? | Consistency between the data and the findings exists | 7-18 |
| 1. Clarity of major themes | Were major themes clearly presented in the findings? | Yes, major themes are clearly identified | 7-18 |
| 1. Clarity of minor themes | Is there a description of diverse cases or discussion of minor themes? | Minor themes are clearly identified and these are related to the major themes | 7-18 |
